# Mononuclear cell composition and activation in blood and mucosal tissue of eosinophilic esophagitis

**DOI:** 10.3389/fimmu.2024.1347259

**Published:** 2024-01-22

**Authors:** Eva Gruden, Melanie Kienzl, Dusica Ristic, Oliver Kindler, David Markus Kaspret, Sophie Theresa Schmid, Julia Kargl, Eva Sturm, Alfred D. Doyle, Benjamin L. Wright, Franziska Baumann-Durchschein, Julia Konrad, Andreas Blesl, Hansjörg Schlager, Rudolf Schicho

**Affiliations:** ^1^ Division of Pharmacology, Otto Loewi Research Center, Medical University of Graz, Graz, Austria; ^2^ Division of Allergy, Asthma, and Clinical Immunology, Mayo Clinic Arizona, Scottsdale, AZ, United States; ^3^ Division of Gastroenterology and Hepatology, Department of Internal Medicine, Medical University of Graz, Graz, Austria

**Keywords:** eosinophilic esophagitis, reflux disease, flow cytometry, T cells, immune cell profile, CD38, PD-1, inducible mouse model

## Abstract

**Introduction:**

Eosinophilic esophagitis (EoE) is a chronic, inflammatory, antigen-driven disease of the esophagus. Tissue EoE pathology has previously been extensively characterized by novel transcriptomics and proteomic platforms, however the majority of surface marker determination and screening has been performed in blood due to mucosal tissue size limitations. While eosinophils, CD4^+^ T cells, mast cells and natural killer (NK) T cells were previously investigated in the context of EoE, an accurate picture of the composition of peripheral blood mononuclear cells (PBMC) and their activation is missing.

**Methods:**

In this study, we aimed to comprehensively analyze the composition of peripheral blood mononuclear cells and their activation using surface marker measurements with multicolor flow cytometry simultaneously in both blood and mucosal tissue of patients with active EoE, inactive EoE, patients with gastroesophageal reflux disease (GERD) and controls. Moreover, we set out to validate our data in co-cultures of PBMC with human primary esophageal epithelial cells and in a novel inducible mouse model of eosinophilic esophagitis, characterized by extensive IL-33 secretion in the esophagus.

**Results:**

Our results indicate that specific PBMC populations are enriched, and that they alter their surface expression of activation markers in mucosal tissue of active EoE. In particular, we observed upregulation of the immunomodulatory molecule CD38 on CD4^+^ T cells and on myeloid cells in biopsies of active EoE. Moreover, we observed significant upregulation of PD-1 on CD4^+^ and myeloid cells, which was even more prominent after corticosteroid treatment. With co-culture experiments we could demonstrate that direct cell contact is needed for PD-1 upregulation on CD4^+^ T cells. Finally, we validated our findings of PD-1 and CD38 upregulation in an inducible mouse model of EoE.

**Discussion:**

Herein we show significant alterations in the PBMC activation profile of patients with active EoE in comparison to inactive EoE, GERD and controls, which could have potential implications for treatment. To our knowledge, this study is the first of its kind expanding the multi-color flow cytometry approach in different patient groups using *in vitro* and *in vivo* translational models.

## 1 Introduction

Eosinophilic esophagitis (EoE) is an immune-mediated disease, characterized by increased infiltration of eosinophils in the esophagus mucosa. EoE is diagnosed in patients presenting with symptoms of esophageal dysfunction, such as vomiting or dysphagia, with esophageal mucosal biopsies demonstrating at least 15 eosinophils per high power field (eos/hpf) (1). What was once considered a rare disease is now, due to its increasing incidence and prevalence, the most common cause of food impaction (2, 3). Interestingly, a recent meta-analysis of EoE incidence and prevalence in Europe suggests discrepancies and underdiagnosis of EoE in Europe when compared to North America, where the clinical awareness and research focus on the disease is more pronounced (4).

The diagnostic delay in EoE still ranges between 1-6 years, which is a concern as longer periods of untreated inflammation are associated with a higher occurrence of esophageal fibrosis and food impaction, leading to decreased quality of life, especially in pediatric patients (5–7). In order to prevent fibrotic complications, early intervention and treatment of ongoing active inflammation is needed (8). Current off-label, approved and investigative treatments for EoE include proton pump inhibitors (PPI), topical (swallowed) corticosteroids and dietary therapy. Moreover, dupilumab - a monoclonal antibody that simultaneously blocks the signaling pathways of IL-4 and IL-13, has recently entered clinical use and has proven to be effective in around 60% of patients (9, 10). Treatment goals in EoE include inducing histological remission (defined as <15 eos/hpf) and relieving symptoms of esophageal dysfunction (8). Unfortunately, neither therapy is universally efficient nor fully curative, mainly due to diverse pathogenic mechanisms (endotypes) driving EoE phenotypes that carry clinical relevance for treatment-responsiveness. Particularly as they are not strictly related to the number of eosinophils in esophageal tissue.

The disease pathogenesis of EoE is primarily mediated by T helper 2 (T_H_2) cells and characterized by increased interleukin (IL)-5, IL-13 and IL-33 levels resulting in eosinophil infiltration and activation (1, 11, 12). Increased immune cell infiltrate is connected with structural alterations of the esophagus such as decreased barrier function and esophageal epithelial cell remodeling as well as hyperproliferation of less mature epithelial subpopulations (13, 14). In fact, a bi-directional crosstalk has previously been described, where co-culture of eosinophils with epithelial cells led to prolonged eosinophil survival and changes in epithelial cell transcriptome (15).

EoE was previously thought to be a component of gastroesophageal reflux disease (GERD). However, it is now recognized to be a separate disease entity with multifactorial pathogenesis (16). It is still thought to be a tissue-centered disease, where inflammation is localized to the esophagus and therefore has to be routinely diagnosed with biopsy (1, 16). As such, patient biopsy material in combination with novel transcriptomic (17) and proteomic platforms (18) has previously identified involvement of diverse immune cell populations such as mast cells (19) and pathogenic T_H_2 cells (11, 20, 21) in EoE pathogenesis. But a systematic analysis of multiple PBMC populations and their surface activation markers in blood and mucosal tissue has so far not been performed.

In this study, we investigated PBMC composition and specific surface activation marker expression in blood and mucosal tissue of patients with active EoE (>15 eos/hpf), inactive EoE (0 eos/hpf), GERD patients (that often share symptoms similar to EoE) (22) and control subjects. We identified increased infiltration of natural killer (NK) T cells, NK cells and myeloid cell populations in mucosal tissue of patients with active EoE and increased CD3^-^CD4^+^ cells in blood of patients with active EoE. Moreover, we observed increased surface expression of CD38, an immunomodulatory receptor and enzyme, on CD4^+^ T cells and myeloid cells in active EoE. Evaluated cell populations and surface activation markers were further investigated and validated using co-culture with primary human esophageal epithelial cells, and in a novel mouse model of inducible EoE. Altogether, the experiments provide a comprehensive analysis of PBMC composition and activation in active EoE compared to inactive EoE and controls.

## 2 Materials and methods

### 2.1 Patient demographics

**Figure 1:**
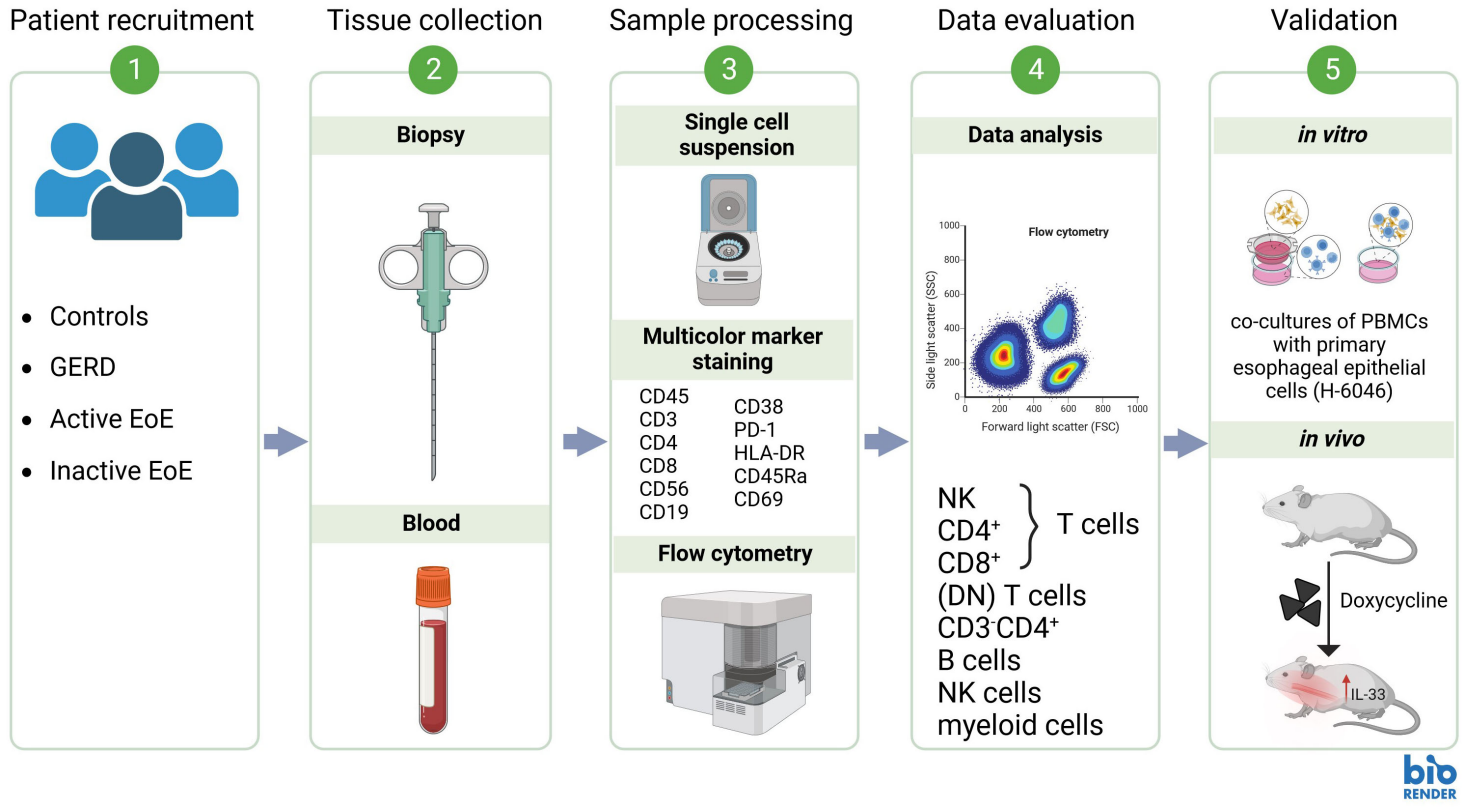
Proposed research workflow.

Age- and sex-matched patients diagnosed with EoE or GERD and control subjects were recruited at the Division of Gastroenterology and Hepatology, University Hospital Graz, from July 2021 to November 2023 (clinical study protocol number: EK# 31-492 ex 18/19). Blood (20 mL) and esophageal mucosal biopsies (proximal and distal segments of the esophagus) were collected and immediately processed for flow cytometry. Two biopsies per patient were immediately fixed in 10% phosphate-buffered formalin for histochemical analysis. Patients with symptomatic and histologically confirmed EoE (>15 eos/hpf) at time of sample collection were grouped as ´Active EoE´. Symptoms of EoE included difficulties swallowing, chest/abdominal pain or heartburn, vomiting or food impaction. Asymptomatic patients with EoE treated with corticosteroids who had 0 eos/hpf at time of sample collection were grouped as ‘inactive EoE’. Patients exhibiting GERD symptoms and with histologically confirmed absence of eosinophils in the esophagus were classified as GERD group. Control individuals were defined as asymptomatic individuals without a history of esophageal pathologies. To avoid bias, the investigators performing the analyses did not take part in the recruitment process and were blinded to diagnosis during analyses of blood and mucosal tissue. Subjects with significant comorbidities, intercurrent illness (such as infections), and pregnant women were excluded from the study. In total, 10 patients with active EoE, 5 patients with inactive EoE, 8 GERD patients, and 22 controls were recruited. Complete clinical characteristics of recruited patients and controls are represented in **Table 1**: Patient characteristics and in **Supplementary Table 1**. Workflow of the research plan is presented in **Figure 1**.

**Table 1:** Patient characteristics.

		Controls	GERD	Active EoE	Inactive EoE	p-value
**N**		22	8	10	5	
**Age (y)**		60 (44,69)	55 (49,71)	38 (32,51)	55 (49,56)	p=0.35
**Gender male n (%)**		11 (50)	5 (63)	8 (73)	4 (80)	p=0.39
**Eosinophils/hpf**		0	0	30 (25,55)	0	
**Treatment (%)**	**PPI**	14	66.7	0	0	
	**Corticosteroid(topical)**	5	0	33	100	
	**NSAID**	5	0	0	0	

Data are presented as median with interquartile range or percentage of total. Statistical differences between groups were analyzed with chi-square test. hpf, high power field; PPI, proton pump inhibitors; NSAID, non-steroidal anti-inflammatory drugs.

### 2.2 Inducible mouse model of eosinophilic esophagitis

The mouse model of inducible EoE – termed iEoE33, was a kind gift of Dr. A. Doyle and Dr. B. Wright (Mayo Clinic, Arizona, USA). In these mice, a secreted form of IL-33 is overexpressed in the esophageal epithelium under tetracycline (Tet) inducible expression (23). The mice were bred and housed in the animal facilities of the Medical University of Graz in accordance with national and international guidelines. All experimental procedures were approved by the Austrian Federal Ministry of Science and Research (protocol number 2021-0.799.497). Male and female mice 8-12 weeks of age were used for the experiments. In order to induce EoE-like pathology, iEoE33 mice and wild-type controls were given doxycycline in water (1 mg/ml in 2% sucrose) for 14 days. After 7 days, doxycycline was changed in order to retain its efficacy as described previously (24). At the experimental end point (day 14), the total body weight of the mice was measured and their esophagi were harvested for further analysis. The mucosal tissue was weighed and further processed into single cell suspensions for flow cytometry.

### 2.3 Flow cytometry

#### 2.3.1 PBMC isolation

PBMC were isolated immediately after collecting blood in Vacuette^®^ EDTA tubes (Greiner-Bio-One, Austria). Whole blood was processed as described previously (25, 26). First, platelet-rich plasma was removed by centrifugation while erythrocytes were removed by dextran sedimentation. PBMC were isolated by density gradient centrifugation (Lympho Spin Medium, pluriSelect) and counted following isolation. The purity and viability of PBMC preparation was greater than 95%. Two million cells per staining panel were used for flow cytometry. PBMC for coculture experiments were isolated from blood of healthy volunteers according to a protocol approved by the Ethics Committee of the Medical University of Graz (17-291 ex 05/06). All participants signed a written informed consent.

#### 2.3.2 Preparation of single cell suspensions

Single cell suspensions of human esophageal mucosal biopsies were prepared according to digestion procedure published for dissected tumor tissue with minor modifications (27, 28). Using surgical scissors, 2 fresh biopsies (proximal and distal) per patient were cut into small pieces, and afterwards digested with DNase I (160 U/mL; Worthington) and collagenase (4.5 U/mL; Worthington) for 20 min at 37°C, while rotating at 750 rpm. After that, the tissue was passed through a mini pluriStrainer^®^ 40 μm strainer, used for small sample volumes, directly in a 5 mL reaction flow cytometry tube. Samples were then resuspended in staining buffer (PBS + 2% FBS), washed with PBS, and used for surface and antigen staining.

Weighed mouse esophagi (minimum 15 mg of weight) were digested similarly and washed with PBS. Immediately after digestion, 20 µL of counting beads (Precision Count Beads™, Biolegend) were added to each sample.

#### 2.3.3 Staining protocol and analysis

To exclude dead cells, PBMC and single cell suspensions from tissue were initially incubated for 20 min in Fixable Viability Dye (FVD) eFluor™ 780 (eBioscience) in PBS at 4°C in the dark. Prior to staining, single cell suspensions were incubated in 1 μg anti-mouse or anti-human TruStain FcX™ (BioLegend, #422304 or #101320, RRID : AB 1574975) for 10 min. Immunostaining was performed for 30 min at 4°C (protected from light) using the pre-mixed antibody panels (**Supplementary Tables 2**, **3**). Cells were washed and fixed in eBioscience™ IC Fixation Buffer (ThermoFisher Scientific, # 00-8222-49) for 10 min at 4°C. Cells were finally washed and resuspended in staining buffer in order to be acquired on a BD LSR FortessaTM flow cytometer with FACSDiva software (BD Biosciences). FlowJo software (Treestar) was used for analysis and compensation. Fluorescence minus-one-samples were used to define gates of cell populations and activation markers (see gating strategy in **Supplementary Figures 1**, **2**).

### 2.4 Histology

Formalin-fixed paraffin-embedded sections of human mucosal esophageal biopsies were cut (5 µm), deparaffinized, and processed for hematoxylin and eosin (H&E) staining or further used for immunohistochemistry. To stain eosinophils, sections were microwaved for 2 x 5-min cycles in 10 mM citrate buffer, and processed by ABC method according to the manufacturer’s protocol (Vectastain ABC kit; Vector Labs; PK-6101). Sections of human biopsies were then incubated with mouse anti-EPX antibody (clone MM25-82.2; 5 µg/ml) followed by treatment with a secondary anti-mouse biotinylated antibody. Antibodies were kindly donated by Dr. Elizabeth Jacobsen, Mayo Clinic Scottsdale, AZ, USA). Images were taken with a high-resolution digital camera (Olympus UC90) and analyzed by Olympus cellSense Standard 1.17 imaging software (Olympus, Vienna, Austria). Contrast, brightness and color balance of images were adjusted using Corel Photo Paint^®^ (Corel Corp.).

### 2.5 Co-culture with human primary esophageal epithelial cells

Human primary esophageal epithelial cells were purchased from Cell Biologics (Chicago, IL, USA; Cat. # H-6046) cultivated in complete human epithelial cell medium provided by the vendor and used until passage seven. For the purpose of indirect co-culture one confluent flask (75 mm^2^) of H-6046 cells was seeded on three separate 12-well transwell plates with inserts (Corning, 12 mm transwell with 0.4 μm pore polycarbonate membrane insert). The inserts were precoated with 0.1% gelatin (15 min, 37 °C). Once cells grew to confluence on the transwell inserts (~48h) the media was replaced with starvation media (human epithelial cell media without supplements and FBS). After 4-12 h of starvation, 1 million isolated PBMC from healthy volunteers were resuspended in starvation media and added in the bottom compartment of the transwell plate. Control PBMC were added to bottom compartments, where no epithelial cells were grown. After 72 h, PBMC were collected from bottom compartments and processed for flow cytometry. For the purpose of direct co-culture, epithelial cells were seeded in 6-well plates. Once confluent, epithelial cells were starved as described previously and allogenic PBMC were added directly to cells for the duration of 72 h. After that, PBMC were collected and processed for flow cytometry.

### 2.6 Statistical analysis

Data are presented as means + standard error of means (SEM). Statistical analyses for experiments was performed using GraphPad Prism 10.0.3 (GraphPad Software). Significant differences between two experimental groups were determined using unpaired or paired Student’s *t-*tests, multiple *t*-tests, one-way or two-way ANOVA. In all cases, a p-value <0.05 was considered significant and represented with one, two or three asterisks when lower than 0.05, 0.01, or 0.001, respectively. Heat maps were created using GraphPad Prism 10.0.3, where normalization of values was performed by taking the lowest data set value as 0% and the highest value as 100%. Principal component analysis (PCA) was conducted and plots generated with R package PCAtools (29).

## 3 Results

### 3.1 Characterization of PBMC composition in blood and mucosal blood of EoE patients and controls

**Figure 2:**
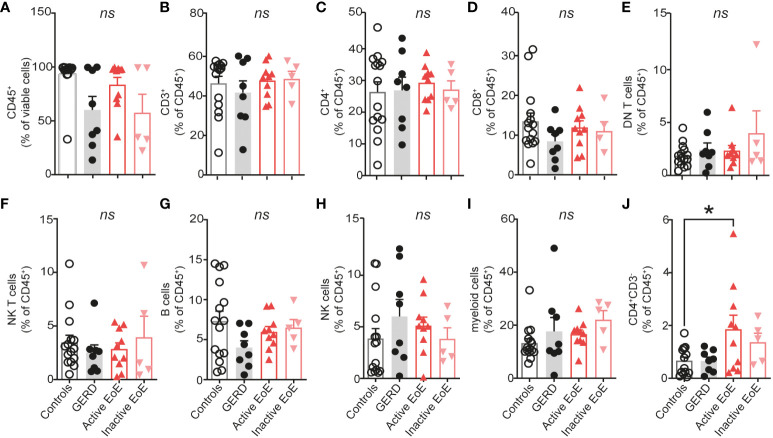
PBMC composition in blood. **(A–J)** Composition of isolated PBMC fraction in blood of donors expressed as percentage of viable or CD45^+^ cells. Data are shown as mean + SEM and statistical differences were assessed by using one-way ANOVA with Tukey’s *post hoc* test *p <.05; *ns*, non-significant.

In order to determine the PBMC composition of whole blood of patients and controls we isolated PBMC from EDTA whole blood of our clinical study participants (**Table 1**). Gating strategy and antibody panel can be found in the **Supplementary Material** section (**Supplementary Figure 1A**, **Supplementary Table 2**). Workflow of our research design is presented in **Figure 1**. Our data shows no significant changes in percentages of major PBMC immune cell populations (**Supplementary Figure 1**, **Figures 2A–I**). However, our flow cytometry screening uncovered a significantly higher proportion of CD3^-^CD4^+^ cells in blood of patients with active EoE compared to control subjects. In our cohort, the percentage of CD3^-^CD4^+^ cells reached approximately 2% of CD45^+^ cells versus 1% in control subjects (**Figure 2J**).

**Figure 3:**
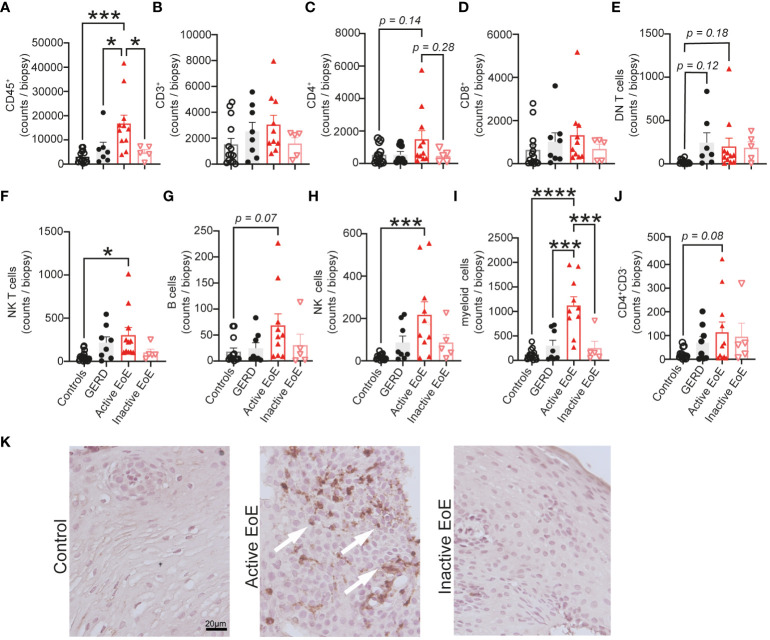
Mononuclear cell composition in esophageal biopsies. **(A–J)** Flow cytometric analysis of single cell suspensions from patient mucosal biopsies. A proximal and distal biopsy were pooled and total counts per biopsy are presented on the graph. **(K)** Representative EPX staining of control, active EoE and inactive EoE (left to right) patient biopsies (calibration bar: 20 µm). EPX positive cells are highlighted by white arrows. Data are shown as mean + SEM and statistical differences were assessed by using one-way ANOVA with Tukey’s *post hoc* test *p <.05; ***p <.001; ****p<.0001.

In order to determine the PBMC composition of esophageal mucosal biopsies, we digested the mucosal tissue into single cell suspensions and analyzed it with multi-color flow cytometry. We observed significantly increased infiltration of CD45^+^ immune cells in esophageal biopsies of patients with active EoE, compared to controls, GERD or inactive EoE (**Figure 3A**). Contrastingly, we did not observe higher infiltration of CD3^+^ T cells in mucosal tissue of active EoE patients (**Figure 3B**) and only trends of increased CD4^+^ T cells (**Figure 3C**), CD8^+^ T cells (**Figure 3D**), and double negative (DN) T cells (**Figure 3E**). Additionally, we observed higher counts of NK T cells (CD3^+^, CD56^+^ cells) (**Figure 3F**) and trends in increased B cells (**Figure 3G**). The most prominent and significant changes of immune cell infiltration in active EoE were observed in the counts of NK cells (**Figure 3H**) and myeloid cells (**Figure 3I**). In contrast to blood, only a trend in increased CD3^-^CD4^+^ cells was observed in mucosal tissue of active EoE (**Figure 3J**). A generally higher (but not significant) immune cell and PBMC infiltration was observed in mucosal tissue of GERD donors compared to controls, indicating increased levels of inflammation.

Immunohistochemistry using a specific eosinophil peroxidase (EPX) antibody was performed on mucosal biopsies from control, active EoE and inactive EoE patients as a marker of eosinophilic inflammation (**Figure 3K**). Our representative images confirm the presence of EPX, one of the major eosinophil granule proteins, specifically in a patient with active EoE.

### 3.2 Activation markers CD38 and PD-1 show major changes in their expression during active EoE

**Figure 4:**
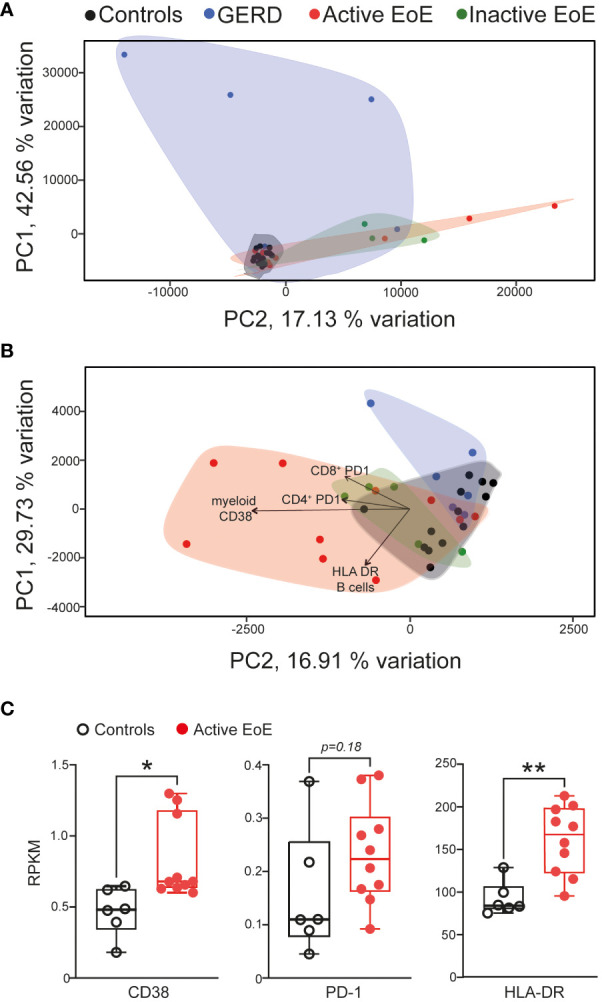
Multivariate analysis (PCA) of activation markers on PBMC populations. **(A, B)** PCA analysis of activation marker expression in different PBMC subsets in blood **(A)** or mucosal tissue **(B)** of controls, GERD, active EoE or inactive EoE patients. **(C)** mRNA expression of CD38, PD-1 and HLA-DR in total transcriptome analysis of control vs active biopsies as adapted and acquired from Sherril et al. (17). N=6-10; Individual reads per kilo base per million (RPKM) data are depicted on top of boxplots showing median and interquartile range as well as minimum and maximum values. Statistical differences were assessed by using unpaired Student´s t-test *p <.05, **p <.01.

Next, a selection of cell activation markers was investigated on the surface of PBMC populations in blood and mucosal tissue in our cohorts. Due to panel and tissue size limitation we focused on investigating expression of CD38 – a receptor and enzyme crucial to immunometabolic pathways and effector functions in most PBMC populations (30), PD-1 – an immune checkpoint protein (31), HLA-DR – Human Leukocyte Antigen – DR isotype involved in antigen presentation (32, 33), CD69 – general early activation marker (34, 35) and CD45RA – a marker of naïve lymphocytes (36). When we performed multivariate analysis (PCA) of chosen surface activation markers, we observed no clear separation of our groups in blood PBMC populations (**Figure 4A**), in contrast to activation markers on populations in esophageal biopsies that showed good separation and stronger changes between our patient groups (**Figure 4B**). In particular, the active EoE group cluster separated much further from controls and inactive EoE patients. The GERD cohort also separates clearly from EoE confirming it as a different disease entity despite some symptom overlap.

The main drivers for the separation in the PCA analysis were CD38 expression on myeloid cells as well as PD-1 surface expression on CD4^+^ and CD8^+^ T cells and HLA-DR expression on B cells (**Figure 4B**). When we compared our major hits to an online available total transcriptome RNA-Seq dataset (17) of active EoE and control patients, we observed that CD38 and HLA-DR expression are significantly higher expressed in the group of active EoE patients (**Figure 4C**), thus, confirming alterations in surface expression in an independent mRNA dataset.

**Figure 5:**
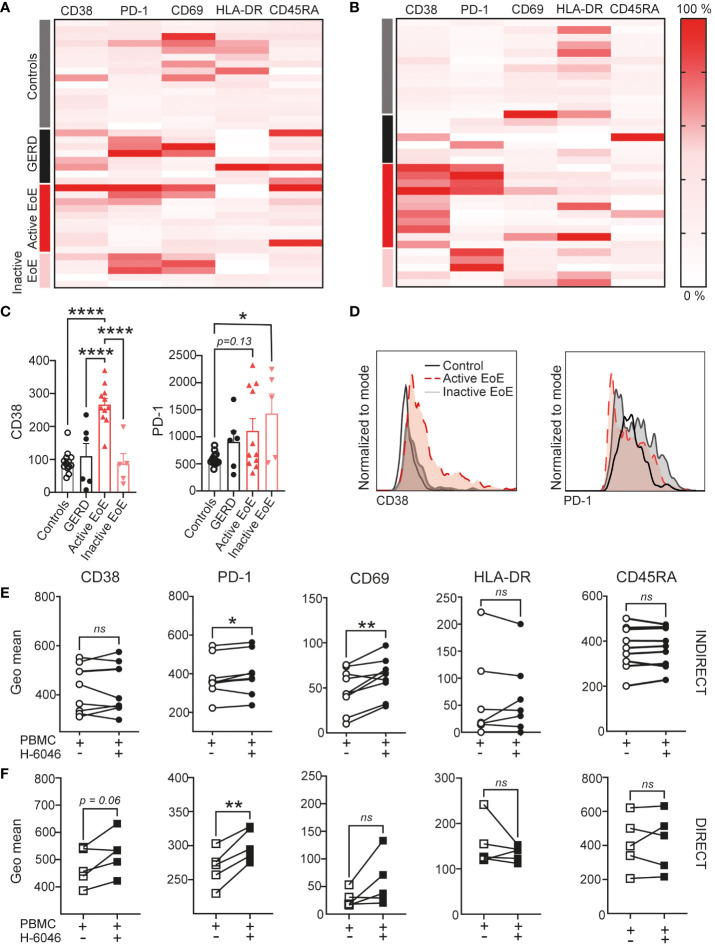
CD4^+^ T cell activation in EoE. Heatmap of surface activation markers expressed on CD4^+^ T cells in blood **(A)** and mucosal tissue **(B)** of our clinical cohort. Each row represents an individual donor and each column a different activation marker. Data within columns is normalized with surface expression being represented by color (pink, lowest expression; dark red, highest expression). **(C)** Bar chart representing measured geo mean intensity of CD38 and PD-1 on CD4^+^ T cells in mucosal tissue of our study subjects. **(D)** An overlay of representative histograms showing CD38 (left) and PD-1 (right) intensity on CD4^+^ T cells in mucosal tissue of a control, active EoE and inactive EoE patient measured on the same day with identical instrument setup. **(E, F)** Surface activation marker expression on CD4^+^ T cells following 72h of indirect **(E)** or direct **(F)** co-culture with human primary esophageal epithelial cells (H-6046). Data are shown as mean + SEM or individual data points. Statistical differences were assessed by using one-way ANOVA with Tukey’s *post hoc* test or paired Student´s t-test *p <.05; **p <.01; ****p<.0001. ns, non significant.

Subsequent detailed analysis of multicolor flow cytometric analysis of activation markers on the surface of CD4^+^ T cells in blood (**Figure 5A**) and mucosal tissue (**Figure 5B**) is represented with heatmaps using column normalization. Our data shows that surface expression of CD38 is significantly upregulated on the surface of CD4^+^ T cells in mucosal tissue of active EoE when compared to controls, GERD and inactive EoE patients (**Figures 5C, D**). Additionally, we observed trends in upregulation of PD-1 on the surface of CD4^+^ T cells, when compared to controls. Interestingly, the upregulation of PD-1 on CD4+ T cells in mucosal tissue of patients with inactive EoE was even higher and statistically significant, when compared to controls (**Figures 5C, D**). To further investigate the up-and down- regulation of surface markers on CD4^+^ T cells in the context of mucosal tissue vs. blood, we performed *in vitro* co-cultures of PBMC (isolated from healthy donors) with primary human esophageal epithelial cells (H-6046). We observed that indirect co-culture conditions are sufficient to upregulate PD-1 and CD69 surface expression on CD4^+^ T cells (**Figure 5E**), while direct co-culture is needed to induce trends in upregulation of CD38 on CD4^+^ T cells (**Figure 5F**).

**Figure 6:**
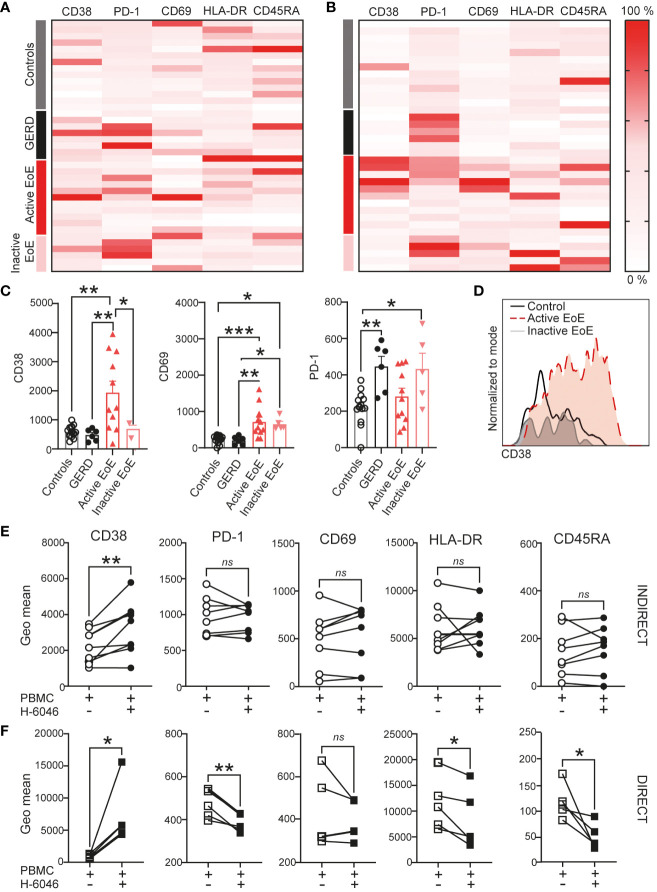
Myeloid cell activation in EoE. Heatmap of surface activation markers expressed on myeloid cells in blood **(A)** and mucosal tissue **(B)** in our clinical cohort. Each row represents an individual donor and each column a different activation marker. Data within columns is normalized with surface expression being represented by color (pink, lowest expression, dark red, highest expression). **(C)** Bar chart representing measured geometric mean intensity of CD38, CD69 and PD-1 on myeloid cells in mucosal tissue of our donors. **(D)** An overlay of representative histograms showing CD38 intensity on myeloid cells in mucosal tissue of a control, active EoE and inactive EoE patient measured on the same day with identical instrument setup. **(E, F)** Surface activation marker expression on myeloid cells following 72h of indirect **(E)** or direct **(F)** co-culture with human primary esophageal epithelial cells (H-6046). Data are shown as mean + SEM or individual data points. Statistical statistical differences were assessed by using one-way ANOVA with Tukey’s *post hoc* test or paired Student´s t-test *p <.05; **p <.01; ***p <.001. ns, non significant.

Since myeloid cells (gated according to size, **Supplementary Figure 1A**) were one of the major drivers of separation in our cohorts, we similarly investigated surface activation markers on myeloid cells in detail. Our heatmaps presenting changes in activation marker surface expression on myeloid cells (**Figures 6A, B**) likewise show major changes between patient groups in mucosal tissue. In **Figure 6C** we show significantly higher CD38 and CD69 expression on the surface of myeloid cells in active EoE. Furthermore, we show that PD-1 is significantly upregulated in GERD donors and in inactive EoE patients. In contrast to CD4^+^ T cells, we could observe that indirect co-culture with primary esophageal epithelial cells are sufficient to upregulate CD38 on the surface of myeloid cells (**Figure 6D**). In contrast, while most markers are not altered following indirect co-culture (**Figure 6E**), our direct co-culture experiment showed that PD-1, HLA-DR and CD45RA are downregulated on myeloid cells when in direct contact with primary human esophageal epithelial cells (**Figure 6F**).

Corresponding analysis of marker expression including heatmaps for blood and mucosal tissue and co-culture investigation for other cell subsets (CD8^+^ T cells, double negative (DN) T cells, NK T cells, NK cells and B cells) can be found in **Supplementary Materials** (**Supplementary Figures 2**-**6**). The only cell population exhibiting trends in activation markers in blood were B cells, where we observed higher CD45RA and lower HLA-DR surface expression on surface of B cells in active EoE patients (**Supplementary Figure 4**).

### 3.3 Changes in PBMC composition and activation can be validated in an inducible mouse model of EoE

**Figure 7:**
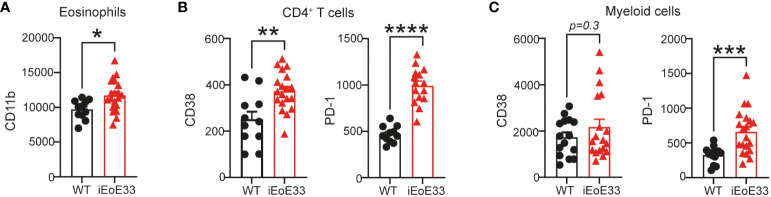
Validation of data in an inducible mouse model of EoE. 8-12-week-old iEoE33 mice or WT littermates were exposed to doxycycline (1 mg/mL in 2% sucrose) for the duration of two weeks. **(A)** Geometric mean of activation marker CD11b surface expression on eosinophils. **(B)** Geometric mean of activation marker CD38 and PD-1 surface expression on CD4^+^ T cells and on myeloid cells **(C)** infiltrating iEoE33 or WT esophagus. Data are shown as individual values + SEM and statistical differences were assessed by using unpaired Student´s t-test *p <.05; **p <.01; ***p <.001; ****p<.0001.

In order to validate our findings of PBMC composition and activation, we utilized a novel inducible mouse model of EoE, where disease pathology is induced under doxycycline exposure leading to increased secretion of the active form of IL-33 in the esophagus (23). This leads to mouse weight loss, thickening of esophagus tissue and clinical characteristics similar to human EoE. When iEoE33 mouse esophagi were analyzed with flow cytometry to deduce the activation state of infiltrating immune cells, we could observe multiple similarities to our patient cohorts. Changes in immune cell composition were accompanied by an altered activation state (**Supplementary Table 3**, **Figure 7**). As such, eosinophils infiltrating in iEoE33 esophagi expressed significantly higher surface expression of CD11b, suggesting higher activation (**Figure 7A**). Additionally, we observed increased CD38 and PD-1 surface expression on CD4^+^ T cells, which was consistent to our patients with active EoE (**Figure 7B**, **Supplementary Figure 1B**). However, we observed no changes in CD38 expression, but a significant increase in PD-1 on the surface of myeloid cells (**Figure 7C**) from WT vs iEoE33 mice, hinting at potential differences in the myeloid cell compartment in this specific mouse model.

## 4 Discussion

In the present study we provide a comprehensive flow cytometric analysis of PBMC in blood and esophageal mucosal tissue of EoE. We describe altered PBMC composition in mucosal tissue and identify CD3^-^CD4^+^ cells to be altered in blood of active EoE patients. Moreover, we show altered surface expression of markers such as CD38 and PD-1 on CD4^+^ T cells and on myeloid cells in active EoE for the first time. Our clinical data was further validated by *in vitro* co-culture systems of PBMC with human primary esophageal epithelial cells and in a novel *in vivo* inducible mouse model of EoE.

EoE is a disease characterized by eosinophilic infiltration and T_H_2 type inflammation. For this reason, researchers designed and carried out clinical studies targeting and depleting eosinophils with the aim of improving EoE pathogenesis. Surprisingly, IL-5 targeting biologics (mepolizumab, reslizumab) and even complete eosinophil depletion with benralizumab (anti- IL5Rα; MESSINA, NCT04543409) or lirentilimab (anti-Siglec-8; KRYPTOS, NCT04322708) in the esophagi of patients with EoE did not lead to complete symptom improvement and clinical remission (37, 38). Additionally, eosinophils were found to be dispensable in an experimental model of EoE characterized with IL-33 overexpression in the esophagus further hinting at pivotal roles of other immune cells (39, 40). Due to this we set out to investigate other immune cell populations infiltrating the esophagus during active EoE.

EoE has long been considered a tissue-centered disease, whose disease severity, symptoms and response to treatment do not reliably correlate to any blood-based biomarkers (e.g. blood eosinophil counts) (41). In line with this, flow cytometric analysis of our cohort does not show significant alterations in percentages of most abundant PBMC populations in blood of active EoE patients. Surprisingly, however, we observed a significantly higher percentage of a minor CD3^-^CD4^+^ population in patients with active EoE, that has previously been directly correlated with lymphocytic variant of hypereosinophilic syndrome (42). To our knowledge, ours is the first report describing a significantly higher proportions of these cells in blood of active EoE patients.

Increased infiltration of certain lymphoid and myeloid populations, such as mast cells, B cells and subsets of CD4^+^ T cells (11, 43, 44) in active EoE has previously been reported. Moreover, an increase in CD11d^+^ invariant NK T cells was previously described in two independent studies of active EoE (45, 46). Our data additionally shows an increase in total NK T cells (gated as viable, CD45^+^, CD3^+^, CD56^+^ single cells) in active EoE mucosal tissue, compared to controls. However, our observations show a similar increase of NK T cells in a group of patients with GERD, suggesting that NK T cell infiltration into the esophagus is not directly related to eosinophilic inflammation. This finding is confirmed by others who discovered higher invariant NK T cell infiltration in laryngopharyngeal reflux (47), showcasing the benefits of including GERD patients to clinical studies investigating EoE pathogenesis. Finally, our data showed significantly higher infiltration of NK cells, which has previously not been reported for EoE. We further validated our PBMC composition data in an inducible mouse model of EoE, in which iEoE33 mice lose weight under doxycycline exposure, and which are characterized by thickening of the esophagus wall accompanied by increased infiltration of eosinophils in the esophagus mucosal tissue (23).

Not just composition, but also activation profile of various immune cell populations can provide information on disease pathogenesis and severity. For this reason, we performed PCA analysis of surface expression measurements of a subset of activation markers (CD38, PD-1, HLA-DR, CD69 and CD45RA) on our immune cell populations. Again, separation of patient groups by analyzing blood samples was poor, but much better in mucosal tissue. The main identified drivers of separation (CD38, HLA-DR and PD-1) between patient groups in mucosal tissue were further confirmed to be increased in active EoE in an independent online repository of RNA-Seq data of tissue control and active EoE biopsies (17).

CD38 is a pleiotropic, ubiquitously expressed receptor and ectoenzyme that is robustly induced during inflammation and infection and is generally considered a drug target in aging and metabolic diseases (48–50). Moreover, in mice, its deficiency has been associated with defects in immune cell infiltration and responses (30). Important for the context of allergic diseases is the fact, that CD38^+^ CD4^+^ T cells were characterized to be hypo-proliferative with a specific bias towards IL-13 secretion (with no change toward IL-4 or IL-5 secretion) (51). IL-13 is described to be crucial for the development of EoE pathology, and it is reversible under glucocorticoid treatment (52, 53). We were therefore especially intrigued to observe a higher expression of CD38 on CD4^+^ T cells in our active EoE patients, which was reduced in patients with inactive EoE responding to glucocorticoid treatment. When we performed co-cultures of isolated PBMC from healthy donors with human primary esophageal epithelial cells, we could observe that direct co-culture is needed to upregulate CD38 on the surface of CD4^+^ T cells. Similar trends of CD38 upregulation (and its reversibility under glucocorticoid treatment) could be additionally observed on myeloid, CD8^+^ T cells and NK T cells. In contrast to CD4^+^ T cells myeloid cells upregulated CD38 on their surface both in direct and indirect co-culture conditions, indicating the sufficiency of epithelial-derived soluble factors to affect CD38-related immunometabolic pathways in myeloid but not in CD4^+^ T cells. The increase of CD38 surface expression was further confirmed *in vivo* on CD4^+^ T cells in tissue of iEoE33 mice, whereas surface expression of CD38 on myeloid cells was not affected in contrast to human active EoE.

Another major hit in our flow cytometric panel was the surface expression of immune checkpoint inhibitor PD-1 – a marker of T cell activation/exhaustion, which is mainly investigated in the context of cancer, but has been reported to be involved in many allergic diseases (54). For example, it was found to be increased on immune cell populations in the nasal mucosa of allergic rhinitis patients (55) and correlated with disease severity and IL-5 levels in chronic rhinosinusitis with nasal polyps (56). Our data shows a trend of increased PD-1 expression in mucosal tissue CD4^+^ T cells of active EoE patients. This finding was further confirmed *in vivo* both on CD4^+^ T cells and myeloid cells, as well as on CD8^+^ T cells (data not shown). Importantly, we discovered an even higher upregulation of PD-1 on the surface of cells (CD4^+^ T cells, myeloid) of patients receiving and responding to glucocorticoid treatment. This fact could be explained by a study, where *in vitro* dexamethasone treatment leads to induction of PD-1 expression in activated mouse and human T cells (57). Moreover, endogenous glucocorticoids (GCs) were reported to be indispensable for PD-1 induction on NK cells (58). To our knowledge, our study is the first reporting increased PD-1 expression on lymphocytes in mucosal tissue following glucocorticoid treatment, which opens new questions especially with increasing rates of oral glucocorticoid prescriptions associated with immune check point inhibitor use in a clinical setting (59, 60), where higher endogenous glucocorticoid levels are associated with worse response rates to immune check point blockade (61). Our findings may be of importance considering that a single trial found that almost 40% of patients under immune check point inhibitor treatment for melanoma receive GCs at least for a short term (60).

In comparison to mucosal tissue, we observed no major changes in surface expression of activation markers in blood PBMC populations. The most prominent (but not significant) changes were observed on the surface of B cells in blood, where patients with active and inactive EoE showed lower levels of HLA-DR on their surface and patients with active EoE showed trends towards higher CD45RA surface expression. Previous studies showed a decrease of the activation marker HLA-DR on B cells following dexamethasone treatment in severe COVID-19 infections and its association with disease severity. Furthermore, patients with sepsis show decreased levels of HLA-DR^+^ B cells, signifying their role during active inflammation (62, 63).

Limitations of our study include the low patient recruitment numbers and the single-center design. Moreover, we were limited in the size and complexity of our panel design (14 colors), therefore we could not investigate additional cell populations and markers. Furthermore, mucosal tissue biopsies were limited in size (approx. 2 mg) and due to this we could not extract enough single cells to design multiple staining panels. Another limitation of our inducible mouse model was, that it relies on expression of high levels of IL-33 and as such, does not include an allergen sensitization component and the involvement of adaptive immunity in the EoE-like pathology.

In conclusion, our study provides an overview of PBMC cell composition and activation utilizing multi-color flow cytometric analysis of blood and mucosal tissue from controls, GERD patients, and active as well as inactive EoE patients. We were able to identify a novel population of CD3^-^CD4^+^ cells to be increased in blood of patients with active EoE. Furthermore, our data suggest that the immunomodulatory marker CD38 as well as immune checkpoint protein PD-1 expression could play a role in EoE pathogenesis. With the finding of increased PD-1 surface expression following corticosteroid treatment our data have potential implications for therapy prescription.

## Data availability statement

The original contributions presented in the study are included in the article/**Supplementary Materials**, further inquiries can be directed to the corresponding author/s.

## Ethics statement

The studies involving humans were approved by Ethics Committee of the Medical University of Graz (17-291 ex 05/06 and EK# 31-492 ex 18/19). The studies were conducted in accordance with the local legislation and institutional requirements. The participants provided their written informed consent to participate in this study. The animal study was approved by Austrian Federal Ministry of Science and Research (protocol number 2021-0.799.497). The study was conducted in accordance with the local legislation and institutional requirements. Written informed consent was obtained from the individual(s) for the publication of any potentially identifiable images or data included in this article.

## Author contributions

EG: Conceptualization, Data curation, Formal Analysis, Investigation, Visualization, Writing – original draft, Writing – review & editing. MK: Conceptualization, Data curation, Investigation, Writing – review & editing. DR: Investigation, Writing – review & editing. OK: Data curation, Formal Analysis, Writing – review & editing. DK: Investigation, Writing – review & editing. SS: Investigation, Writing – review & editing. JKa: Conceptualization, Writing – review & editing. ES: Writing – review & editing. AD: Methodology, Resources, Writing – review & editing. BW: Methodology, Resources, Writing – review & editing. FB-D: Writing – review & editing. JKo: Writing – review & editing. AB: Writing – review & editing. HS: Writing – review & editing. RS: Conceptualization, Funding acquisition, Methodology, Resources, Supervision, Writing – review & editing.
