# Supplementary material for: Mononuclear cell composition and activation in blood and mucosal tissue of eosinophilic esophagitis
Source: Front Immunol. 2024 Jan 22;15:1347259. doi: 10.3389/fimmu.2024.1347259 (PMC10839056; doi:10.3389/fimmu.2024.1347259)
Supplement: Supplementary file 1 [file DataSheet_1.docx]

**Supplementary material:** **Mononuclear cell composition and activation in blood and mucosal tissue of eosinophilic esophagitis**

Eva Gruden^1*^, Melanie Kienzl^1^, Dusica Ristic^1^, Oliver Kindler^1^, David Markus Kaspret^1^, Sophie Theresa Schmid^1^, Julia Kargl^1^, Eva Sturm^1^, Alfred D. Doyle^2^, Benjamin L. Wright^2^, Franziska Baumann-Durchschein^3^, Julia Konrad^3^, Andreas Blesl^3^, Hansjörg Schlager^3^, Rudolf Schicho^1^

^1^Division of Pharmacology, Otto Loewi Research Center, Medical University of Graz, Graz, Austria

^2^Division of Allergy, Asthma, and Clinical Immunology, Mayo Clinic Arizona, Scottsdale, Arizona, USA

^3^Division of Gastroenterology and Hepatology, Department of Internal Medicine, Medical University of Graz, Graz, Austria

**Table 1 : Additional clinical characteristics of active EoE patients**

| Patient code | Eos / hpf |
| --- | --- |
| #4 | 50 |
| #8 | 70 |
| #10 | 25 |
| #16 | 30 |
| #20 | 80 |
| #21 | 25 |
| #37 | 30 |
| #43 | 50 |
| #45 | 25 |
| #46 | 25 |

**Table 2: Human flow cytometry panel**

| Antibody | Dilution | Clone | Company | Catalogue # | RRID |
| --- | --- | --- | --- | --- | --- |
| CD45-AF700 | 1:200 | HI30 | Biolegend | 304024 | AB_493761 |
| CD3-APC | 1:80 | UCHT1 | Biolegend | 304024 | AB_314065 |
| CD4-BUV395 | 1:40 | SK3 | BD | 563550 | AB_2738273 |
| CD8-BV650 | 1:100 | SK1 | Biolegend | 344730 | AB_2564510 |
| CD56-PE | 1:160 | HCD56 | Biolegend | 318306 | AB_604101 |
| Epcam-PE/Dazzle594 | 1:33 | 9C4 | Biolegend | 324231 | AB_2564301 |
| CD45Ra-BV785 | 1:100 | HI100 | BD | 564442 | AB_2738810 |
| CD38-PE-Cy7 | 1:80 | HB-7 | Biolegend | 356607 | AB_2561903 |
| HLA-DR-BV510 | 1:40 | L-243 | Biolegend | 307645 | AB_2561948 |
| CD69-BV605 | 1:40 | FN50 | Biolegend | 310938 | AB_2562307 |
| PD-1-BUV737 | 1:67 | EH12.1 | BD | 565299 | AB_2739167 |
| CD19-BV421 | 1:100 | HIB19 | Biolegend | 302234 | AB_10897802 |

**Table 3: mouse flow cytometry panel**

| Antibody | Dilution | Clone | Company | Catalogue # | RRID |
| --- | --- | --- | --- | --- | --- |
| CD45-AF700 | 1:400 | 30-F11 | Biolegend | 103128 | AB_493715 |
| CD3-BUV395 | 1:80 | 145-2C11 | BD | 563565 | AB_2738278 |
| CD4-BUV496 | 1:160 | GK1.5 | BD | 564667 | AB_2722549 |
| CD8-PerCPCy5.5 | 1:160 | 53-6.7 | Biolegend | 100734 | AB_2075239 |
| Siglec-F-PE | 1:80 | 1RNM44N | BD | 562068 | AB_10896143 |
| NKp46-BV510 | 1:40 | 29A1.4 | Biolegend | 137623 | AB_2563290 |
| CD19-BV650 | 1:57 | 6D5 | Biolegend | 115541 | AB_11204087 |
| Ly-6G-PE/Dazzle^TM^ | 1:333 | 1A8 | Biolegend | 127648 | AB_2566318 |
| PD-L1-PE-Cy7 | 1:160 | 10F.9G2 | Biolegend | 124314 | AB_10643573 |
| CD11b-BUV737 | 1:160 | M1/70 | BD | 612801 | AB_2870128 |
| CD38-APC | 1:40 | 90 | Biolegend | 102712 | AB_312933 |
| PD-1-BV421 | 1:160 | 29F.1A12 | Biolegend | 135217 | AB_2561447 |

**
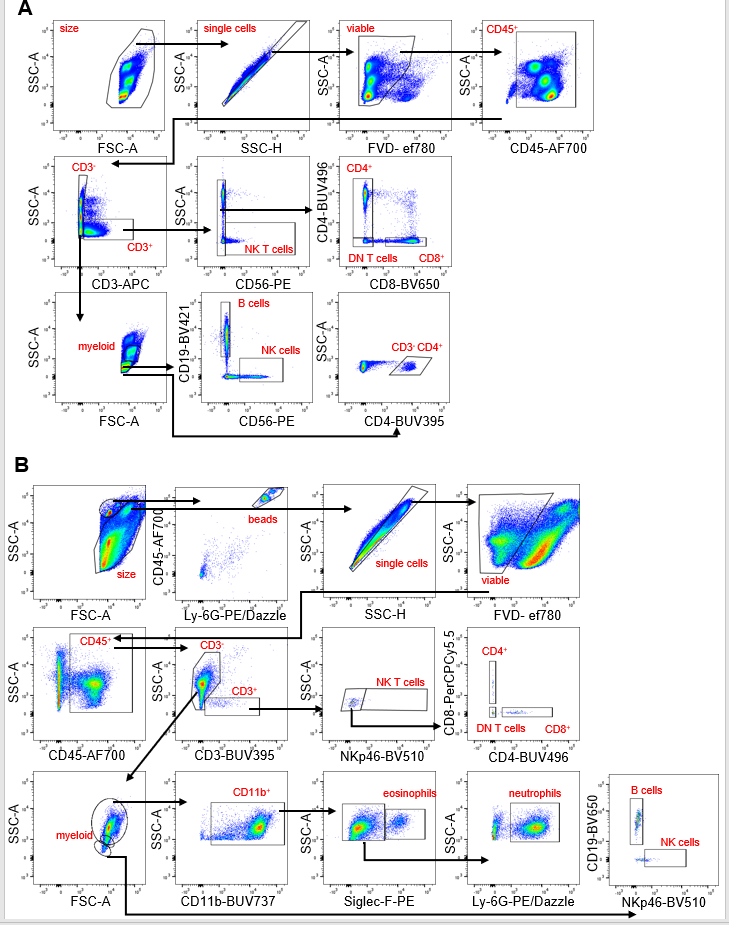
**

**Figure 1: Flow cytometric gating strategies.** Schematic presentation of the human **(A)** and mouse **(B)** gating strategy for analysis of single cell suspensions of blood PBMC and esophagus tissue. Leukocytes (CD45^+^) were pre-gated for single cells. Dead cells were excluded. **(A)** T cells were determined as CD45^+^/CD3^+^; NK T cells as CD45^+^/CD3^+^/CD56^+^; CD8^+^ T cells as CD45^+^/CD3^+^/CD8^+^; CD4^+^ T cells as CD45^+^/CD3^+^/CD4^+^ , double negative (DN) T cells as CD45^+^/CD3^+^/CD4^-^/CD8^-^; myeloid as CD45^+^/CD3^-^/high SSC; B cells as CD45^+^/CD3^-^/low SSC/ CD56^-^/CD19^+^/; NK cells as CD45^+^/CD3^-^/low SSC/CD19^-^/CD56^+^ and CD3^-^CD4^+^ cells as CD45^+^/CD3^-^/low SSC/CD4^+^. **(B)** Counting beads were gated according to size (high SSC) and high double intensity in AF700 and PE Dazzle channels. T cells were determined as CD45^+^/CD3^+^; NK T cells as CD45^+^/CD3^+^/NKp46^+^; CD8^+^ T cells as CD45^+^/CD3^+^/CD8^+^; CD4^+^ T cells as CD45^+^/CD3^+^/CD4^+^ , double negative (DN) T cells as CD45^+^/CD3^+^/CD4^-^/CD8^-^; myeloid as CD45^+^/CD3^-^/intermediate SSC; eosinophils as CD45^+^/CD3^-^/high SSC/CD11b^+^/Siglec-F^+^; neutrophils as CD45^+^/CD3^-^/high SSC/CD11b^+^/Ly-6G^+^; B cells as CD45^+^/CD3^-^/low SSC/NKp46^-^/CD19^+^; NK cells as CD45^+^/CD3^-^/low SSC/CD19^-^/NKp46^+^.


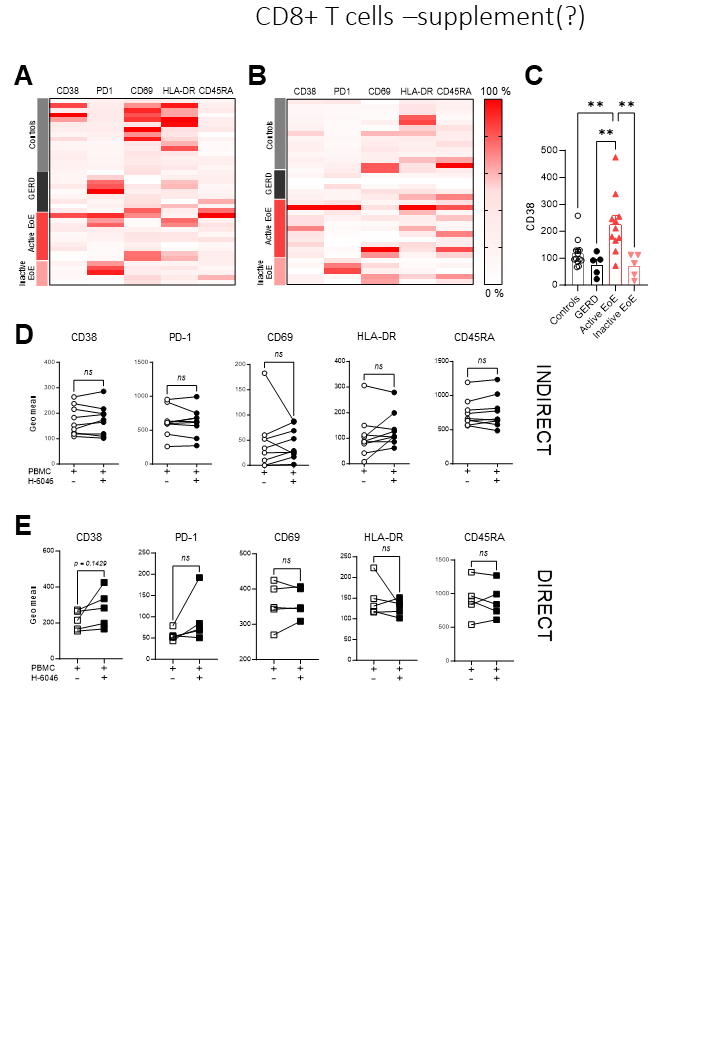


**Figure 2: CD8^+^ T cell activation in EoE.** Heatmap of surface activation markers expressed on CD8^+^ T cells in blood **(A)** and tissue **(B)** in our clinical cohort. Each row represents an individual donor and each column a different activation marker. Data within columns is normalized with surface expression being represented by color (pink, lowest expression; dark red, highest expression). **(C)** Bar chart representing measured geometric mean intensity of CD38 on CD8^+^ T cells in tissue of our donors. **(D-E)** Surface activation marker expression on CD8^+^ T cells following 72h of indirect (**D**) or direct (**E**) co-culture with human primary esophageal epithelial cells (H-6046). Data are shown as mean + SEM or individual values and statistical differences were assessed by using one-way ANOVA with Tukey’s post hoc test or paired Student´s t-test **p < .01.


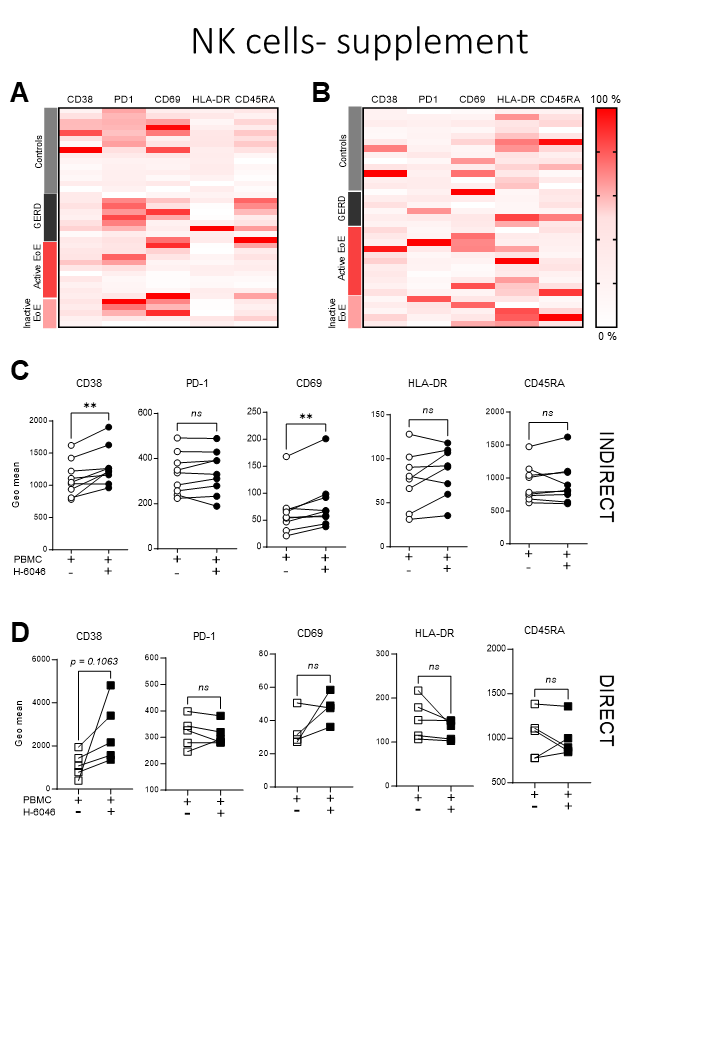


**Figure 3: NK T cell activation in EoE.** Heatmap of surface activation markers expressed on NK T cells in blood **(A)** and tissue **(B)** in our clinical cohort. Each row represents an individual donor and each column a different activation marker. Data within columns is normalized with surface expression being represented by color (pink, lowest expression; dark red, highest expression). **(C)** Bar chart representing measured geometric mean intensity of CD38 on NK T cells in tissue of our donors. **(D-E)** Surface activation marker expression on NK T cells following 72h of indirect (**D**) or direct (**E**) co-culture with human primary esophageal epithelial cells (H-6046). Data are shown as mean + SEM or individual values and statistical differences were assessed by using one-way ANOVA with Tukey’s post hoc test or paired Student´s t-test *p < .05; **p < .01; ***p < .001.

**
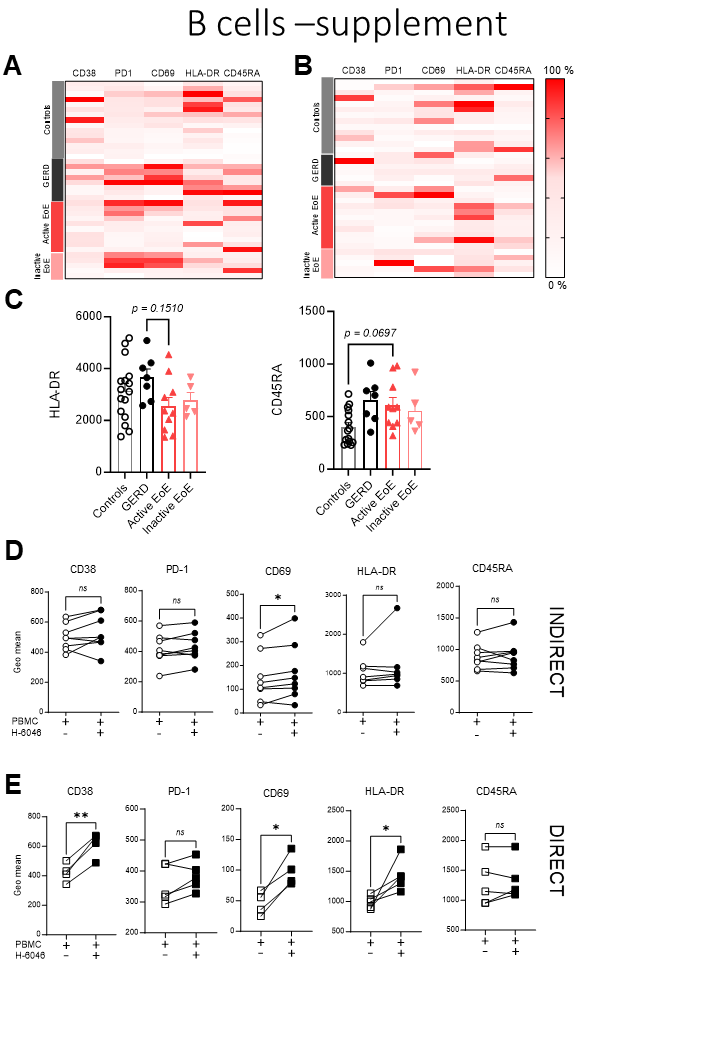
**

**Figure 4: B cell activation in EoE.** Heatmap of surface activation markers expressed on B cells in blood **(A)** and tissue **(B)** in our clinical cohort. Each row represents an individual donor and each column a different activation marker. Data within columns is normalized with surface expression being represented by color (pink, lowest expression; dark red, highest expression). **(C)** Bar chart representing measured geometric mean intensity of HLA-DR and CD45Ra on B cells in blood of our donors. **(D-E)** Surface activation marker expression on B cells following 72h of indirect (**D**) or direct (**E**) co-culture with human primary esophageal epithelial cells (H-6046). Data are shown as mean + SEM and statistical differences were assessed by using one-way ANOVA with Tukey’s post hoc test or paired Student´s t-test *p < .05; **p < .01.

**
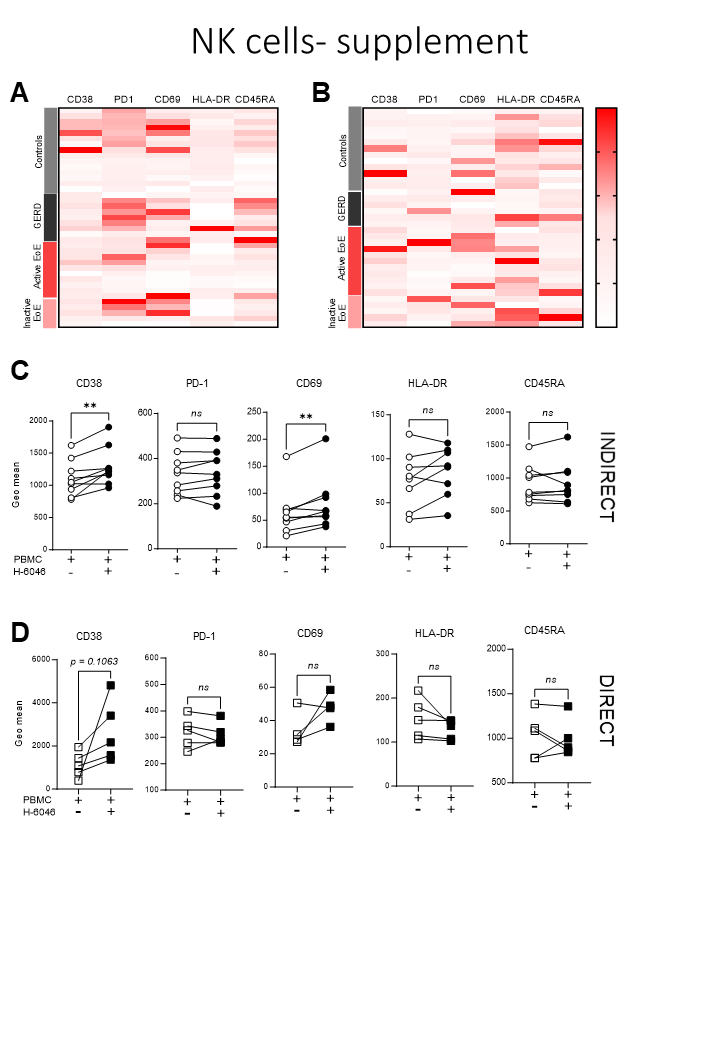
**

**Figure 5: NK cell activation in EoE.** Heatmap of surface activation markers expressed on NK cells in blood **(A)** and tissue **(B)** in our clinical cohort. Each row represents an individual donor and each column a different activation marker. Data within columns is normalized with surface expression being represented by color (pink, lowest expression; dark red, highest expression). **(C-D)** Surface activation marker expression on NK cells following 72h of indirect (**C**) or direct (**D**) co-culture with human primary esophageal epithelial cells (H-6046). Data are shown as individual values and statistical differences were assessed by using one-way ANOVA with Tukey’s post hoc test or paired Student´s t-test **p < .01.


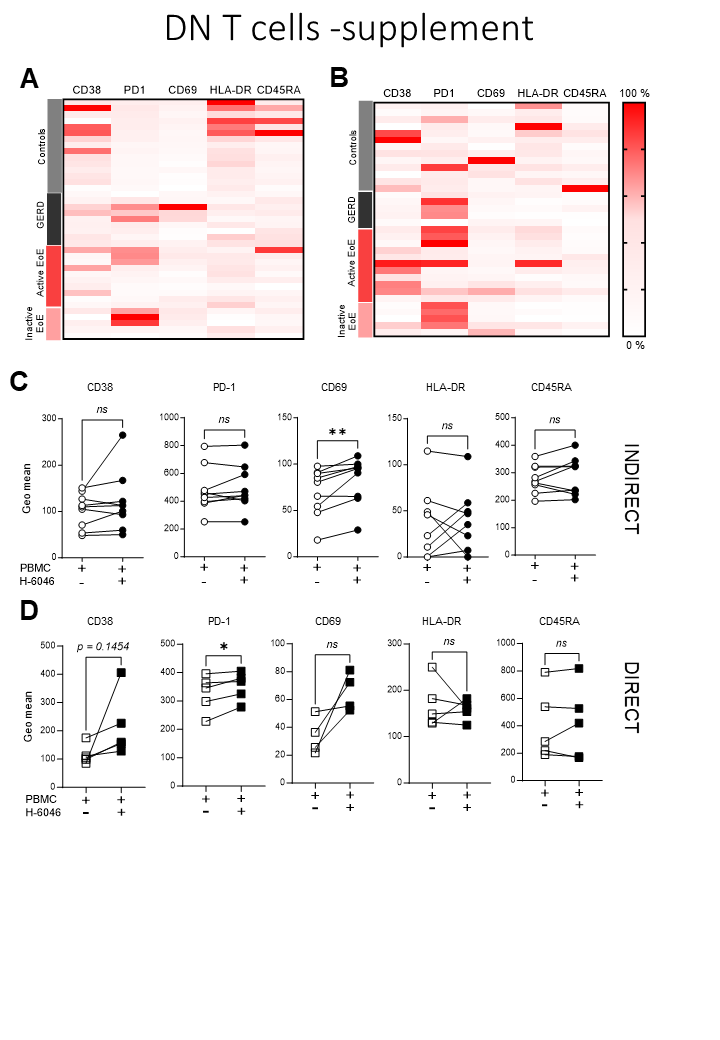


**Figure 6: DN T cell activation in EoE.** Heatmap of surface activation markers expressed on DN T cells in blood **(A)** and tissue **(B)** in our clinical cohort. Each row represents an individual donor and each column a different activation marker. Data within columns is normalized with surface expression being represented by color (pink, lowest expression; dark red, highest expression). **(C-D)** Surface activation marker expression on DN T cells following 72h of indirect **(C)** or direct **(D)** co-culture with human primary esophageal epithelial cells (H-6046). Data are shown as individual values and statistical differences were assessed by using one-way ANOVA with Tukey’s post hoc test or paired Student´s t-test *p < .05; **p < .01.
